# Outcome Predictive Value of Serum Ferritin in ICU Patients with Long ICU Stay

**DOI:** 10.3390/medicina57010001

**Published:** 2020-12-22

**Authors:** Daniel Rusu, Mihaela Blaj, Irina Ristescu, Emilia Patrascanu, Laura Gavril, Olguța Lungu, Ianis Siriopol, Iulian Buzincu, Ioana Grigoraș

**Affiliations:** 1 Anaesthesia—Intensive Care Unit Department, Grigore T Popa University of Medicine and Pharmacy, 700115 Iași, Romania; rusu.daniel.ro@gmail.com (D.R.); anca.ristescu@umfiasi.ro (I.R.); emiliap79@yahoo.com (E.P.); laura-gabriela.gavril@umfiasi.ro (L.G.); Olg-d@yahoo.com (O.L.); ianis.siriopol@gmail.com (I.S.); iulian_s_buzincu@d.umfiasi.ro (I.B.); ioana.grigoras.ro@gmail.com (I.G.); 2 Anaesthesia and Intensive Care Department, Regional Institute of Oncology, 700483 Iași, Romania; 3 Anaesthesia and Intensive Care Department, “Sf. Spiridon” University Hospital, 700111 Iasi, Romania

**Keywords:** iron, ferritin, inflammation, ICU, SOFA score, outcome

## Abstract

*Background and Objectives:* The simplified interpretation of serum ferritin levels, according to which low ferritin levels indicate iron deficiency and high levels indicate hemochromatosis is obsolete, as in the presence of inflammation serum ferritin levels, no longer correlate with iron stores. However, further data are needed to interpret serum ferritin levels correctly in patients with ongoing inflammation. Our study aimed to assess serum iron and ferritin dynamics in patients with long intensive care unit (ICU) stay and the possible correlations with organ dysfunction progression and outcome. *Materials and Methods:* We conducted a prospective study in a university hospital ICU over six months. All patients with an ICU length-of-stay of more than seven days were enrolled. Collected data included: demographics, Sequential Organ Failure Assessment (SOFA) score, admission, weekly serum iron and ferritin levels, ICU length-of-stay and outcome. Interactions between organ dysfunction progression and serum iron and ferritin levels changes were investigated. Outcome predictive value of serum ferritin was assessed. *Results:* Seventy-two patients with a mean ICU length-of-stay of 15 (4.4) days were enrolled in the study. The average age of patients was 62 (16.8) years. There were no significant differences between survivors (39 patients, 54%) and nonsurvivors (33 patients, 46%) regarding demographics, serum iron and ferritin levels and SOFA score on ICU admission. Over time, serum iron levels remained normal or low, while serum ferritin levels statedly increased in all patients. Serum ferritin increase was higher in nonsurvivors than survivors. There was a significant positive correlation between SOFA score and serum ferritin (r = 0.7, 95% CI for r = 0.64 to 0.76, *p* < 0.01). The predictive outcome accuracy of serum ferritin was similar to the SOFA score. *Conclusions:* In patients with prolonged ICU stay, serum ferritin dynamics reflects organ dysfunction progression and parallels SOFA score in terms of outcome predictive accuracy.

## 1. Introduction

Progressions made in disease management and intensive care unit (ICU) innovations have been leading to an increased number of critically ill patients that are being balanced out during acute illness. However, after the initial injury, some of these patients will experience persistent organ dysfunctions, increased susceptibility to infections, general weakness, neuropathy, myopathy, nutritional deficiencies, muscle wasting, anaemia and complex metabolic and immunological disturbances, requiring long-term intensive care [1]. These are the patients with long ICU stay or so-called chronically critically ill [1]. Patients with long ICU stay are a growing challenge for physicians, as they represent up to 10% of all ICU admissions and have a mortality rate as high as 50% [2,3]. Moreover, a study performed in elderly patients found that 1-year mortality rate increased with increasing ICU length of stay [4]. The hallmark of patients with long ICU stay is ongoing inflammation following an initial inflammatory insult [1,5]. While some pro- and anti-inflammatory cytokines have been extensively reviewed, further information is required to better understand the role of other key molecules in persistent inflammation. Among these molecules is serum ferritin.

Ferritin role in iron metabolism is well known [6]. However, the traditional approach in which low ferritin levels indicate iron deficiency and high levels indicate hemochromatosis needs to be revised [7]. One argument is that serum ferritin cut-offs values for diagnosing iron disorders are based on obsolete data [7], and another is that in the presence of inflammation serum ferritin levels no longer correlate with iron availability [8,9]. A recent systematic review undertaken to summarise the evidence for ferritin as a diagnostic biomarker for iron stores revealed that mean ferritin concentration was 82 mcg/L in patients with iron depletion (38 studies, 1023 participants), 381 mcg/L in patients with normal iron stores (38 studies, 1549 participants) and close to 500 mcg/L in patients with iron overload [10]. In conclusion, there is a definite need for further data about serum iron and ferritin dynamics in patients with long ICU stay that have both alterations in iron metabolism and persistent inflammation. 

Our study aimed to evaluate serum iron and ferritin dynamics in critically ill patients with more than seven days of ICU stay and to investigate the correlations between organ dysfunction progression and serum ferritin and iron changes over time. Outcome predictive value of serum ferritin levels in patients with more than seven days of ICU stay was assessed. 

## 2. Materials and Methods

### 2.1. Design and Settings

We performed a prospective non-interventional study to evaluate serum iron and ferritin dynamics in critically ill patients with a long ICU stay. Long ICU stay was defined as more than seven days of critical care. Correlations between serum iron and ferritin, organ dysfunction progression and outcome were explored. The study was performed at the Clinical Emergency Hospital Sf. Spiridon Iasi, a tertiary care university hospital. Enrolled patients were followed-up from ICU admission until hospital discharge or in-hospital death. 

All subjects/legal representatives gave their informed consent for inclusion in the study. The study was conducted in accordance with the Declaration of Helsinki, and the protocol was approved by the Ethics Committee of the Clinical Emergency Hospital Sf. Spiridon Iasi (45/24.11.2020—duplicate).

### 2.2. Participants

Participants were recruited from the critically ill patients admitted to ICU over six months. During this period, all ICU admissions were scrutinised to identify patients expected to have an extended ICU stay. Patients diagnosed with acute respiratory distress syndrome, acute myocardial infarction, severe pancreatitis, septic shock, surgical complications (anastomotic leak with surgical site infection) or multiple severe comorbidities were supposed to need more than seven days of critical care and were enrolled in the study. Patients with acute or chronic kidney disease who were dialysed during ICU stay, and those who were discharged, transferred to other facilities or died during the first week of their ICU stay were excluded. 

### 2.3. Variables

Demographics (age, gender, ICU admission type) were recorded for all patients. 

Serum iron and ferritin were determined on ICU admission and hospital discharge and weekly between these two time-points. Serum iron and ferritin were measured by spectrophotometry and electrochemiluminescence (ECLIA), respectively, using a Cobas^®^ 8000 analyser (Roche Diagnostics, Mannheim, Germany). For serum iron, our laboratory reference range was 37–145 mcg/dL in women and 59–158 mcg/dL in men. For serum ferritin, the reference range was 30–400 mcg/L for males and 13–150 mcg/L for females. 

Organ dysfunction progression was assessed by calculation the Sequential Organ Failure Assessment (SOFA) score. SOFA score was calculated on ICU admission and subsequently in parallel with serum iron and ferritin measurements. Finally, the ICU length-of-stay and in-hospital outcome were recorded for every studied patient. 

### 2.4. Statistical Analysis

MedCalc Statistical Software version 19.1.7 (MedCalc Software Ltd., Ostend, Belgium; https://www.medcalc.org; 2020) was used for statistical analysis. Variables were tested for normality using histograms. Continuous variables were summarised as mean and standard deviation (sd) and categorical variables as number and percentage (%). A student t-test was used to compare continuous variables, and χ2 or Fisher’s exact test to compare categorical variables. A Pearson correlation test was used to calculate the correlation coefficient between serum ferritin and SOFA score. Outcome predictive accuracy of serum ferritin was compared to SOFA score using the pairwise comparison of receiver operating characteristic (ROC) curves. For all analyses, a *p* value <0.05 was considered statistically significant.

## 3. Results

**Figure 1:**
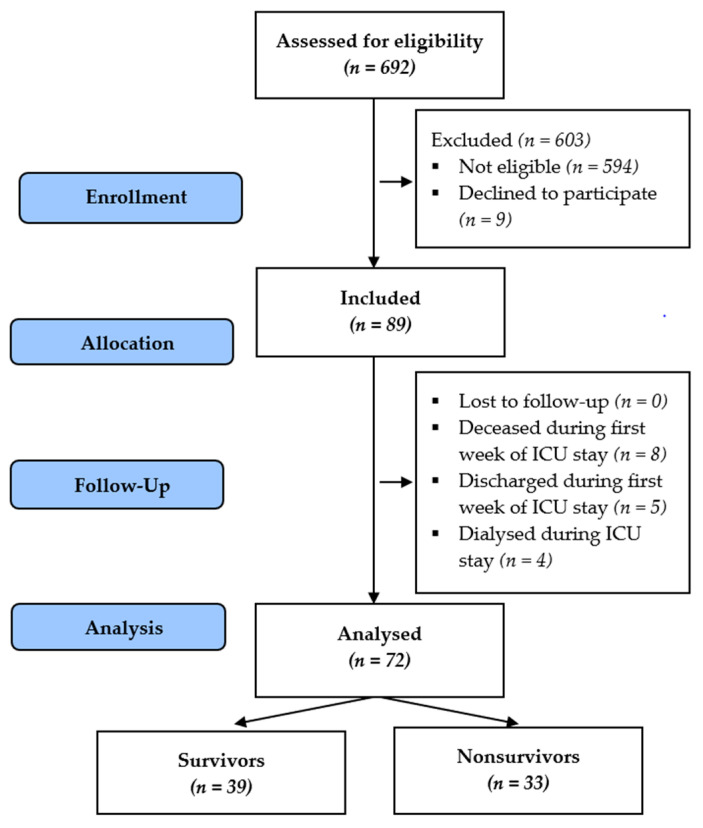
Flow chart of study cohort selection.

Over six months, we identified 89 critically ill patients that fulfilled the inclusion criteria and consented to study enrolment. Out of these patients, 17 were excluded based on exclusion criteria. The remaining 72 patients were included in the final analysis. A flow chart of study cohort selection is presented in Figure 1.

Out of the 72 patients, 46 (64%) were males and 26 (36%) females. The average age of patients was 62 (16.8) years, and the mean ICU length-of-stay was 15 (4.4) days. Forty-six (64%) patients were admitted to the ICU for surgical reasons and 26 (36%) patients for medical conditions. Mean serum iron and ferritin levels on ICU admission were 57 (7.3) mcg/dL and 459 (31.9) mcg/L, respectively. Mean SOFA score on admission was 3.6 (1). The mortality rate among study patients was 46% (33 patients). We found no statistically significant differences between survivors and nonsurvivors regarding demographics, SOFA score, serum iron and ferritin levels on ICU admission. Study patients’ characteristics are presented in Table 1.

**Figure 2:**
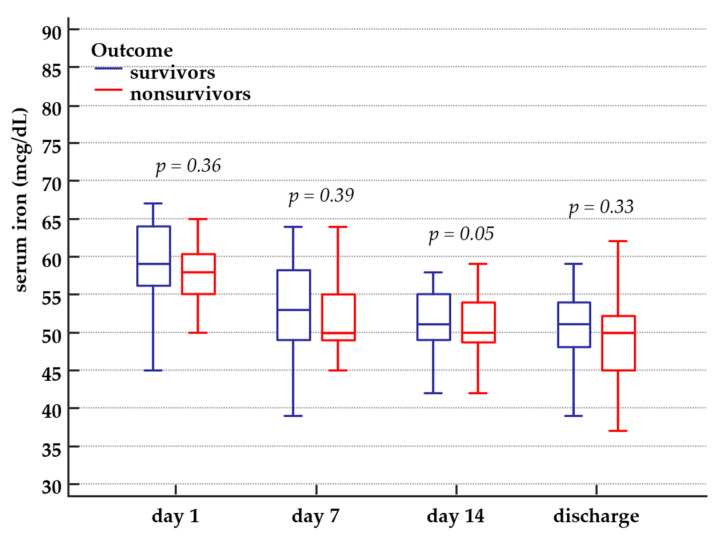
Serum iron dynamics in survivors and nonsurvivors. Serum iron minimum and maximum value, interquartile range and median value in survivors and nonsurvivors, from ICU day 1 until hospital discharge or in-hospital death.

Serum iron levels were in the reference range or decreased on ICU admission and further decreased during hospitalisation. Although overall serum iron decrease was more pronounced in nonsurvivors than survivors, we did not find any significant difference over time between serum iron levels of survivors and nonsurvivors (Table 1). Serum iron dynamics in survivors and nonsurvivors is presented in Figure 2. 

**Figure 3:**
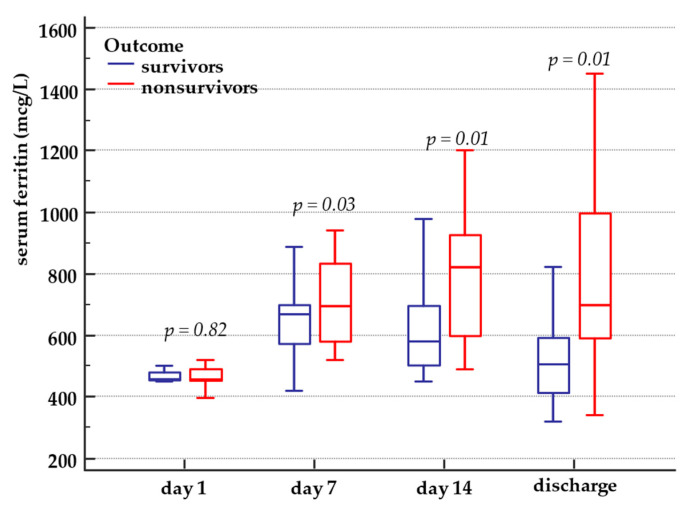
Serum ferritin dynamics in survivors and nonsurvivors. Serum ferritin minimum and maximum value, interquartile range and median value in survivors and nonsurvivors, from ICU day 1 until hospital discharge or in-hospital death.

Serum ferritin levels on ICU admission were higher than the upper value of the reference range in the entire study population. Serum ferritin further increased during ICU stay in all patients and reached significantly higher values in nonsurvivors than survivors (Table 1). Serum ferritin dynamics in survivors and nonsurvivors is presented in Figure 3.

**Figure 4:**
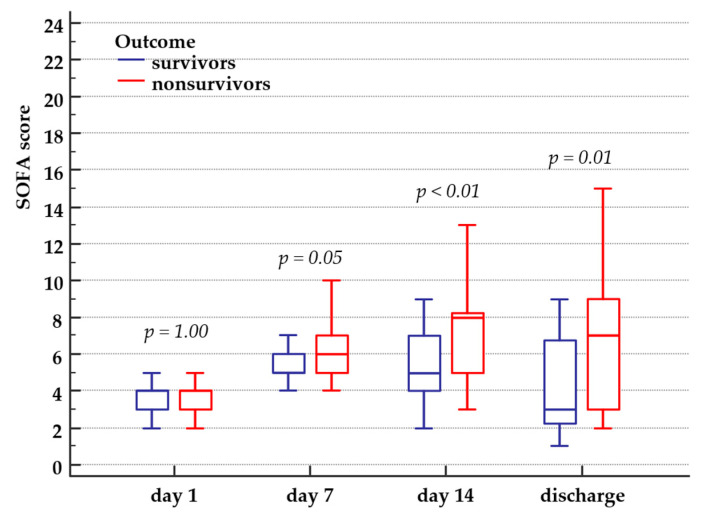
SOFA score in survivors and nonsurvivors. SOFA score minimum and maximum value, interquartile range and median value in survivors and nonsurvivors, from ICU day 1 until hospital discharge or in-hospital death.

As expected, the SOFA score was significantly higher over time in nonsurvivors than in survivors (Figure 4). 

**Figure 5:**
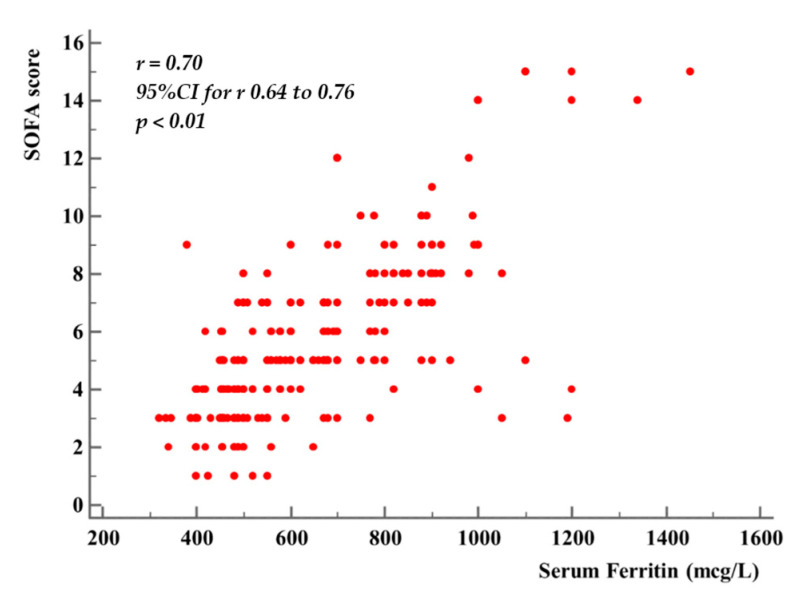
Relation between serum Ferritin and SOFA score.

The mean value of worst SOFA score was 8.4 (3) in nonsurvivors and 6.4 (3) in survivors (*p* < 0.01). Organ dysfunction progression, as expressed by SOFA score increase, was accompanied by serum ferritin increase, while serum iron levels remained normal or decreased. We found a significant positive correlation between SOFA score and serum ferritin (*r* = 0.7, 95% CI for *r* = 0.64 to 0.76, *p* < 0.01) (Figure 5). 

**Figure 6:**
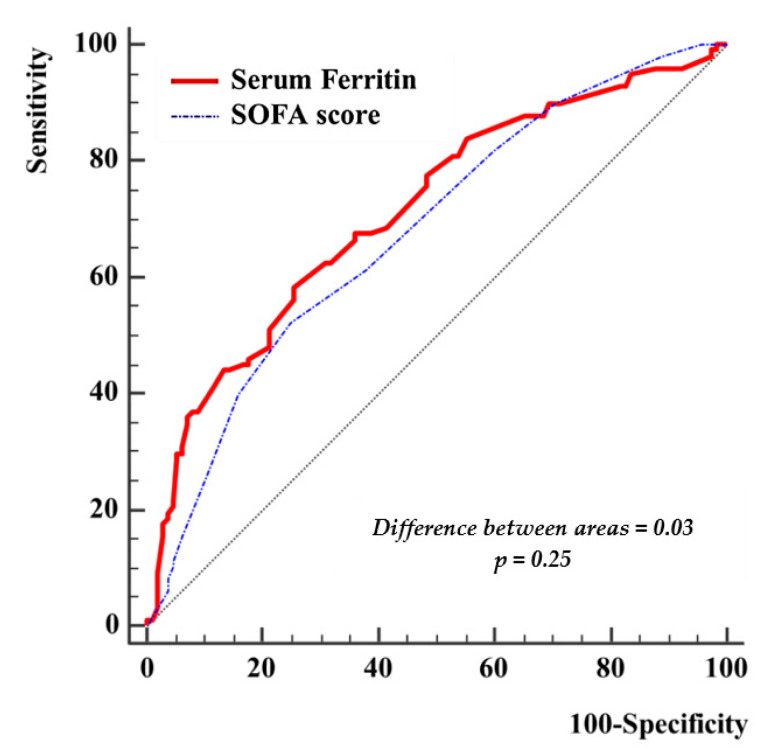
Pairwise comparison of receiver operating characteristic (ROC) curves for serum Ferritin and SOFA score.

The area under the ROC curve (AUROC) for SOFA score was 0.68 (95% CI 0.61–0.74) and for serum ferritin was 0.71 (95% CI 0.65 to 0.77). The pairwise comparison of ROC curves for SOFA score and serum ferritin revealed the same accuracy for outcome prediction (the difference between AUROC 0.03; 95% CI −0.02 to 0.10; *p* = 0.25) (Figure 6). 

## 4. Discussion

Serum ferritin concentration results from the leakage of tissue ferritin, an intracellular iron storage protein shell with a molecular weight of 450 kDa, containing heavy and light subunits [11]. While tissue ferritin plays a role in intracellular iron handling, serum ferritin is believed to be a key mediator of inflammation. The level of ferritin in plasma represents the balance between its secretion and its clearance, mainly in the liver [11]. When inflammation is present, various factors interfere with the synthesis and clearance of ferritin, thereby increasing serum ferritin levels, while mobilisation of iron from the reticuloendothelial system is decreased [11]. Serum ferritin is upregulated by circulating cytokines (e.g., IL-1 and TNF), hypoxia–ischaemia and oxidative stress [9,12]. The potential mechanisms responsible for increased serum ferritin levels in critically ill patients with extended ICU stay are the enhanced systemic inflammatory response, tissue damage and cell lysis, increase ferritin synthesis and decrease ferritin clearance due to liver impairment. Patients with acute or chronic conditions may also play an important role, as abnormal serum ferritin levels have been previously linked to an increased number of diseases: inflammatory and infective disorders (ARDS, Sepsis), malignancy, cardiovascular diseases (Myocardial infarction, Coronary artery disease, Hypertension, Atherosclerosis), hepatic disorders (Cirrhosis, Fatty liver disease), neurological disorders (Stroke), metabolic disorders (Type 2 Diabetes mellitus, Metabolic syndrome) and immune disorders (Rheumatoid arthritis, Systemic lupus erythematosus) [11]. In our study, we analysed the evolution of the inflammatory state of patients by looking at the complications of critical illness developed over time. SOFA score was calculated in dynamics to assess these complications, as this score describes quantitively and objectively the degree of organ dysfunction [13]. We found similar patterns in SOFA score and serum ferritin dynamics and a strong positive correlation between them. Our data suggest that serum ferritin level is not only linked to the presence of inflammation, as previously reported [14], but also mimics inflammation changes. 

A systematic review summarised data about serum ferritin levels in patients with different conditions and with or without iron overload (10). According to this paper, mean serum ferritin value was 500 mcg/L in the absence of iron overload and over 1000 mcg/L in the presence of iron overload [10] for most of the conditions analysed. These data also support our hypothesis that serum ferritin levels were influenced by changes in patient’s inflammatory status (in our study, mean serum ferritin level was increased but remained all-time under 1000 mcg/L). 

The outcome prognostic value of serum ferritin has been previously investigated in different populations [15,16,17,18,19,20]. Serum ferritin was a prognostic marker of a poor outcome in the Sharkey’s [15], Elliot’s [16], Garcia’s [17], Carcillo’s [18] and Dahan [19] studies, while in the study by Pavlou [20], ferritin increase was not correlated with increased mortality. In our study, we found that serum ferritin has a prognostic value, similar to the SOFA score. There was no significant difference in serum ferritin levels on ICU admission between survivors and nonsurvivors as they had similar disease severity (similar SOFA score). However, with critical illness progression, we noticed higher mean SOFA scores and serum ferritin levels in nonsurvivors than in survivors. 

### Study Strengths and Limitations

Our study addressed ICU patients with long ICU stay, a category of patients known to have persistent inflammation. To our knowledge, serum ferritin levels have not been intensively analysed in this population. Moreover, in our study, serum ferritin levels are measured throughout ICU hospitalisation and compared to organ dysfunction progression, assessed with SOFA score. The predictive outcome accuracy of serum ferritin is compared to a well-studied indicator of prognosis in critically ill patients: the SOFA score. This approach is an original one. However, our study also has some limitations. The study was performed in a single centre. The study cohort included a heterogeneous population of medical and surgical patients of different ages and with different conditions who required long ICU care. The cohort may be affected by selection bias, as a probability sampling method was used to select participants (patients expected to have long ICU stay). The cohort size was limited, as the study was performed during a short period (six months). The manner in which serum iron and ferritin were measured may impact the quality of study findings as the time-frame between two consecutive determinations may be considered too long to reflect their true dynamics during ICU stay.

## 5. Conclusions

In critically ill patients with long ICU stay, serum iron levels are normal or low on ICU admission and remain normal or decreased throughout hospitalisation. On the other hand, serum ferritin levels parallel the progression of inflammation and organ dysfunctions, the highest values being found in patients with the worst outcome. Serum ferritin has a predictive outcome accuracy similar to SOFA score.

## Figures and Tables

**Table 1:** Patients’ characteristics.

Variable	All Patients *n =* 72	Survivors *n =* 39 (54%)	Non-Survivors *n =* 33 (46%)	*p* Value
Demographics				
Age (years)	62.5 (16.8)	64.3 (13.7)	60.2 (19.8)	0.32
Gender				
▪ males	46 (64%)	22 (56%)	24 (73%)	0.15
▪ females	26 (36%)	17 (44%)	9 (27%)	0.15
ICU admission type				
▪ surgery	46 (63.9%)	27 (69.2%)	19 (57.6%)	0.56
▪ medical	26 (36.1%)	12 (30.8%)	14 (42.4%)	0.57
Serum Iron (mcg/dL)				
▪ day 1	57 (7.3)	57.8 (7.0)	56.2 (7.5)	0.36
▪ day 7	51.3 (8.2)	52.1 (7.7)	50.4 (8.8)	0.39
▪ day 14	49.1 (5.3)	51.4 (5.2)	46.8 (5.4)	0.05
▪ discharge	49.9 (5.7)	50.5 (5.4)	49.1 (6.1)	0.33
▪ largest decrease by	8 (3.0)	8 (2.5)	9 (3.6)	0.04
Serum Ferritin (mcg/L)				
▪ day 1	459 (31.9)	458 (31.5)	460 (32.9)	0.82
▪ day 7	623 (121.5)	534 (104.7)	718 (132.4)	0.03
▪ day 14	708 (207.7)	635 (183.3)	794 (206.7)	0.01
▪ discharge	658 (253.9)	536 (190.8)	773 (269.6)	0.01
▪ largest increase by	323 (200)	250 (155)	411 (214)	0.01
SOFA score				
▪ day 1	3.7 (1.0)	3.7 (1.1)	3.7 (0.9)	1.00
▪ day 7	5.9 (1.8)	5.6 (1.7)	6.4 (1.8)	0.05
▪ day 14	6.5 (3.1)	5.7 (2.9)	7.6 (3.0)	<0.01
▪ discharge	5.8 (4.0)	4.7 (3.8)	7.1 (3.9)	0.01
▪ worst	7.3 (3.1)	6.4 (3.0)	8.4 (3.0)	<0.01
ICU stay (days)	15 (4.4)	15 (4.9)	14 (3.7)	0.26

Table 1 legend: Data are given as *n* (%) or as mean (sd) as appropriate. Serum iron largest decrease: baseline value (day 1)—lowest value measured from day 7 to discharge. Serum ferritin largest increase: highest value measured from day 7 to discharge—baseline value (day 1). SOFA: Sequential Organ Failure Assessment Score.

## Data Availability

The data presented in this study are available on request from the corresponding author.
